# Organizational commitment and associated factors among health professionals working in public hospitals of southwestern Oromia, Ethiopia

**DOI:** 10.1186/s12913-023-09167-3

**Published:** 2023-02-21

**Authors:** Bizunesh Fantahun, Endalkachew Dellie, Nigusu Worku, Ayal Debie

**Affiliations:** 1 Department of public health, College of Medicine and Health Sciences, Samara university, Semera, Ethiopia; 2 Department of Health Systems and Policy, Institute of Public Health, College of Medicine and Health Sciences, University of Gondar, Gondar, Ethiopia; 3 P. O. Box 196, Gondar, Ethiopia

**Keywords:** Organizational commitment, Health professionals, Public hospitals, Ethiopia

## Abstract

**Introduction:**

Organizational commitment refers to the extent to which employees identify with and are involved with a given organization. It is an important variable for healthcare organizations to consider since it acts as a predictor of job satisfaction, organizational efficiency and effectiveness, health professionals’ absenteeism, and turnover. However, there is a knowledge gap in the health sector about workplace factors that are associated with healthcare provider commitment to their organization. Thus, this study aimed to assess organizational commitment and associated factors among health professionals working in public hospitals in the southwestern Oromia region, Ethiopia.

**Methods:**

A facility-based analytical cross-sectional study was conducted from March 30 to April 30, 2021. A multistage sampling technique was employed to select 545 health professionals from public health facilities. Data were collected using a structured self-administered questionnaire. simple and multiple linear regression analyses were employed to assess the association between organizational commitment and explanatory variables after checking the assumptions of factor analysis and linear regression. The statistical significance was declared at a p-value of < 0.05 and Adjusted Odds Ratio (AOR) with a 95% Confidence Interval (CI).

**Results:**

Health professionals’ organizational commitment percentage mean score was 48.8% (95% CI: 47.39, 50.24). A higher level of organizational commitment was associated to satisfaction with recognition, work climate, supervisor support, and workload. Besides, good practice of transformational and transactional leadership styles and employee empowerment are significantly associated with high organizational commitment.

**Conclusion:**

The overall level of organizational commitment is a bit low. To improve the organizational commitment of health professionals, hospital managers, and healthcare policy-makers need to develop and institutionalize evidence-based satisfaction strategies, practice good leadership styles and empower healthcare providers on the job.

## Background

Organizational commitment refers to the relative strength of an individual’s identification and involvement with the overall employing organization and not with a department or specific workgroup [1]. Organizational commitment also means complying with the aims and objectives of the organization, organizational principles, rules and norms, and volunteering for their survival [2].

Meyer and Allen theorized three types of organizational commitment in 1984: affective, continuance, and normative commitment [3]. Affective commitment is related to an employee’s emotional connection, identification, and participation in the organization. Normative commitment is about employees’ feelings of responsibility to the organization, while continuance commitment is associated with perceived costs related to exiting the organization [3, 4].

According to studies on the issue, several factors such as; work climate, recognition, supervisor support, workload, leadership styles, and individual and job characteristics related variables were the most significant factors that determine employees’ commitment to their organizations [5, 6].

A high level of employee organizational commitment has several implications for healthcare organizations, including a strong desire to achieve organizational goals, a strong desire to stay in the organization, improved organizational performance, increased motivation, belongingness, and attachment to their organization, increased effectiveness and efficiency of their organization, improved job satisfaction, reduced turnover, burnout, and staff absenteeism [7–9]. On the contrary, having poor organizational commitment increases the occurrence of medical errors and poses a threat to patient safety [10].

Researchers found that employees committed to their organization provided a high quality of care [9], had better performance [11], ensured patient safety [12], and continuous routine operations [13]. However, various findings showed organizational commitment among healthcare professionals is low. In Saudi Arabia and Egypt, the magnitude of organizational commitment was reported to be 57.3% and 63.9%, respectively [14, 15]. Similarly, studies on the issue in different parts of Ethiopia showed results ranging from 64.81 to 88.06% [7, 16, 17].

Considerable studies focused on organizational commitment in the field of nursing [18–23]. However, organizational commitment gaps are affecting other healthcare professions as well and there is a shortage of knowledge in the health sector on workplace factors that are associated with employees’ commitment to their organization. Moreover, variables such as leadership styles and health professionals’ empowerment that might significantly influence employees’ organizational commitment have not yet been assessed in previous studies in Ethiopia. Therefore, this study set out to address workplace factors that influence the organizational commitment of healthcare professionals.

Thus, the study fills the knowledge gap in organizational commitment in the health sector and provides evidence for making decisions that are appropriate to the context. Besides, it helps managers and policymakers better understand the underlying issue and implement strategies that improve healthcare providers’ commitment to their organization.

## Method and materials

### Study design and setting

A facility-based analytical cross-sectional study design was employed to assess the organizational commitment of healthcare professionals working in the southwestern Oromia region public health facilities, in Ethiopia from March 30 to April 30, 2021. In this part of the region, there are seven administrative zones, of which, we have chosen three zones, namely Buno Bedele, Ilubabor, and Jimma zones randomly. Bedele, Metu, and Jimma are the administrative towns of the three zones, respectively. The towns are 480, 600, and 354 km away from Addis Ababa, respectively.

According to the 2019 projected population report of Ethiopia, the three zones had a total population of 5,095,050. There are 15 public hospitals in these zones among these hospitals; one found in Jimma town is a specialized hospital, the other found in Metu town namely Karl hospital is a referral hospital and the rest of the public hospitals are primary hospitals. According to the information obtained from Oromia regional health bureau in 2020, there were 2,577 health professionals working in the public hospitals of the three zones.

All health professionals working in public hospitals in the southwestern Oromia region were the source population, whereas health professionals working in randomly selected public hospitals were the study population.

Health professionals who have been working for at least 6 months in the selected public hospitals were included in the study, whereas volunteers and part-time workers were excluded.

### Sample size determination and sampling procedure

The sample size was determined by using a single population mean estimation formula (n = (Zα/2)^2^ * δ^2^/d^2^). A confidence level of 95% and a margin of error(d) of 1% were used. In a similar study at Jimma University specialized hospital, in Ethiopia, a standard deviation (σ) of 8.22 was used [17]. By considering design effect 2 and 10% non-response rate the final sample size was 572.

**Fig. 1:**
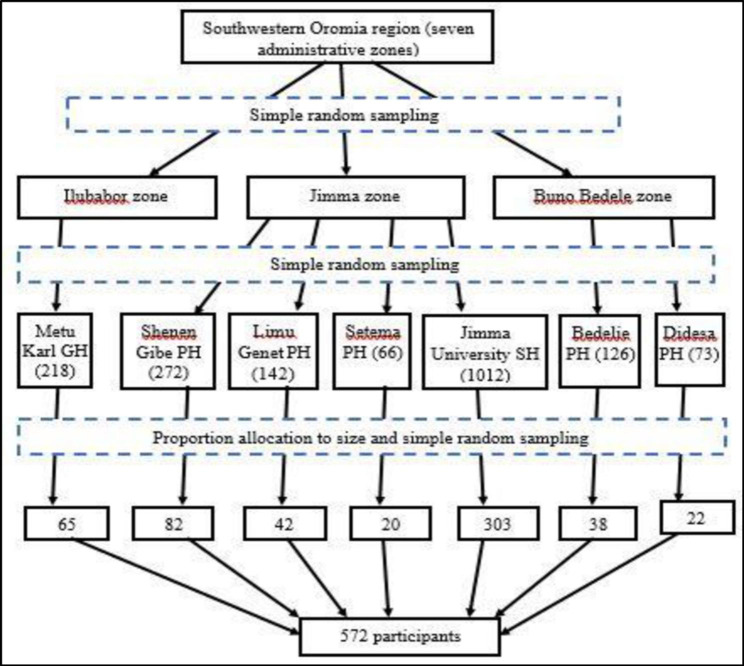
Schematic presentation of sampling procedure **Note**: PH: Primary Hospital; GH: General Hospital and SH: Specialized Hospital

A multi-stage with a simple random sampling technique was used. Initially, out of the seven administrative zones in southwestern Oromia, three zones: Buno Bedele, Ilubabor and Jimma zones were selected by lottery method. Second, seven government hospitals were chosen from the three selected zones using a simple random sampling method. The calculated sample size was then proportionally allocated to each selected hospital according to the number of health professionals they had. Finally, study participants were selected using simple random sampling from the health professionals’ payroll forms (Fig. 1).

### Variables and measurement

#### Organizational commitment

Is the degree of health professionals’ attachment, loyalty, and identification with their organization. Its overall level of organizational commitment was measured by 10 items with a 5-point Likert scale (1 = strongly disagree to 5 = strongly agree) [24]. The mean score was converted to a percentage of the scale mean score (%SM). This value ranges from “0%” to “100%”, and was calculated using the formula: $\text{\%} \text{S}\text{M}=\frac{\text{A}\text{c}\text{t}\text{u}\text{a}\text{l} \text{s}\text{c}\text{o}\text{r}\text{e} - \text{s}\text{c}\text{o}\text{r}\text{e} \text{m}\text{i}\text{n}\text{i}\text{m}\text{u}\text{m}}{\text{s}\text{c}\text{o}\text{r}\text{e} \text{m}\text{a}\text{x}\text{i}\text{m}\text{u}\text{m} ? \text{s}\text{c}\text{o}\text{r}\text{e} \text{m}\text{i}\text{n}\text{i}\text{m}\text{u}\text{m}}\text{*}100$ [25]. The composite scale of organizational commitment score was created from the three factors that emerged during the factor analysis of organizational commitment items. This score was used as a continuous dependent variable during linear regression.

#### Job satisfaction

Is the degree of positive or negative views workers has about their job or job experience. It was measured with four dimensions (perceived recognition (5 items), perceived working climate (10 items), perceived supervisor support (4 items), and perceived workload (3 items)) consisting of 22 items of five-point Likert scales [26].

#### Perceived recognition

The health professionals’ feeling toward the praise they get for doing a good job. It was measured with five items on a 5-point Likert scale [26].

#### Perceived working climate

The quality of both physical attributes and the degree to which the working environment provides meaningful work. It was measured by using ten items each scoring a 5-point Likert scale [26].

#### Perceived supervisor support

Is the supervisors’ responsibility both in preventing and solving employee problems. It was measured by using four items each scoring a 5-point Likert scale [26].

#### Perceived workload

It describes the participant’s work requirement, the amount of time and resources for this requirement. It is measured by using three items each scored 5-point Likert scale [26].

#### Perceived organizational support

It refers to the extent to which employees see that organizations recognize their contribution and care about their well-being. It was measured by four items which were measured in a 5-point Likert type; factor analysis was used to explore the factor structure of the scale [27].

#### Perceived leadership style

This is the perception of HPs regarding how their immediate leader treats them. It was measured with two dimensions consisting of seven items which were created on factor analysis, to be measured on a 5-point Likert scale [28].

#### Perceived transformational leadership style

The perception of health professionals about how their immediate leader influence and inspire them. It was measured by using five items each scoring a 5-point Likert scale [28].

#### Perceived transactional leadership style

The perception of health professionals about how their immediate leader responds or reacts to problems/situations. It was measured by using two items each scoring a 5-point Likert scale [28].

#### Employee empowerment

It describes the feeling of health professionals having proportionate power in their organization. It was measured by using five items each scored on a 5-point Likert scale [29].

### Data collection tools and procedures

Data were collected using self-administered structured questionnaires adapted from different literature and have 6 parts. Part one sociodemographic and economic data with 12 items, part two job satisfaction data comprised 22 items, and part three perceived organizational support questionnaire with 8 items [9]. Part four OC was measured with 24 items which were adapted from questionnaires developed and validated by Meyer and Allen [24], and part five perceived leadership style measure with 9 items adapted from the Multifactor Leadership Questionnaire (MLQ) [28]. Except for the sociodemographic and economic data, all parts will be measured on a 5-point Likert scale ranging from strongly disagree = 1, to strongly agree = 5.

Three public health officers and two BSc nurses for data collection and three public health officer lecturers for supervision were employed. Each data collector collected data from health professionals working in their zone. Supervisors and principal investigators were responsible for routine supervision.

### Data quality control

To ensure consistency, the questionnaires were initially prepared in English and translated into the local language (Afan Oromo and Amharic) and finally back into English. One day of training was given to data collectors and supervisors before the data collection. To ensure the study’s internal validity, the tool was pre-tested with 5% of the sample outside the study area and modification was made before actual data collection. The overall internal consistency of the tools was checked using Cronbach’s alpha reliability test, and the score for the items of each component was greater than 0.79. Moreover, each item was qualitatively evaluated by all authors for readability, meaning, clarity and feasibility related to study participants. To test the discriminatory validity of the item scales that measure each dimension of the constructs of the variable, a principal component analysis was employed. Items with a loading coefficient of less than 0.30 were excluded from further analysis. Because of their weak discriminatory ability with the items of the other constructs, some items were excluded from the subsequent analysis. In the end, a single scale is created by averaging items that fulfill the discriminant validity for each of the variables.

### Data management and analysis

The data were cleaned and checked for consistency, coded, and entered into Epi Info version 7 software, and exported to Stata version 14 statistical software for analysis. Recoding, computing, ranking, and counting were performed before data analysis. All assumptions of factor analysis were checked. The normality of the data was evaluated by performing the Shapiro-Wilk test, and the result indicates evidence of both the normality of the data and the absence of outliers (W = 0.94, P-value = 0.32). The linearity of the data was assessed by generating a scatter plot and all of the points in the plot fall along a straight line. The Variance Inflation Factors (VIF) were used to test for multicollinearity, and all the values were below 10. Homoscedasticity was assessed by generating a plot of standard residual against standard predicted values of the dependent variable in regression models through which the random patterned distribution and independence of error terms were assessed.

Simple linear regression analysis was done to identify candidate variables for multiple linear regression analysis and a p-value of ≤ 0.20 was used to select these variables. Factors predicting organizational commitment were differentiated by using multiple linear regression analysis at a significance level of p-value < 0.05 and a 95% CI.

## Results

### Sociodemographic characteristics of respondents

A total of 545 health professionals participated in this study, with a response rate of 95.30%. More than half of the respondents (57.61%) were males. Nearly two out of the three respondents were married (62.57%) and about 43.67% were nurses in their profession. The median age of the participants was 30 years, Inter Quartile Range (IQR = 7). The median net monthly salary of Health professionals was 6,500 Birr (IQR = 2,900) (Table 1).

**Table 1:** Sociodemographic characteristics of Health professionals working in public hospitals of SW Oromia, 2021 (n = 545)

Variables	Categories	Frequency	Percentage
Sex	Female	231	42.39%
	Male	314	57.61%
Marital status	Married	341	62.57%
	Unmarried	204	37.43%
Profession	Nurse	238	43. 67%
	Midwifery	85	15.59%
	Laboratory professional	78	14.31%
	Pharmacist	64	11.74%
	Doctor	80	14.68%
Working unit/ department	IPD	246	45.14%
	OPD	161	29.54%
	Laboratory	75	13.76%
	Pharmacy	61	11.19%
	Emergency	55	10.09%
Educational status	Diploma	102	18.75%
	Bachelor degree	357	65.63%
	Masters and above	85	15.63%
Type of position	Managerial	95	17.46%
	Non-managerial	449	82.54%
Living house	Within the compound	74	13.6%
	An outside the compound	470	86.4%

### Job satisfaction

Among job satisfaction dimensions, the highest percentage mean score, 53.2%, was observed in the category of working climate, and the lowest score, 44.6%, was in the supervisor support component. (Table 2).

**Table 2:** Magnitude of job satisfaction of health professionals working in public hospitals of southwestern Oromia, 2021 (n = 545)

Emerged factors	Mean raw score ± SD	%SM
Work climate	3.13 ± 0.89	53.2%
Recognition	3.04 ± 0.93	51%
Supervisor support	2.80 ± 0.87	44.6%
Workload	2.96 ± 0.79	48.9%

### Leadership style, organizational support, and employee empowerment

Regarding leadership styles, 46.65% and 46.9% percentage mean score was observed for transformational and transactional leadership styles, respectively. The percentage mean score of organizational support and employee empowerment was 48.75% and 52.85%, respectively (Table 3).

**Table 3:** Magnitude of leadership styles, supervisor support, and employee empowerment of health professionals working in public hospitals of southwestern Oromia, 2021 (n = 545)

Emerged factors	Mean raw score ± SD	%SM
Transformational leadership style	2.87 ± 0.92	46.65%
Transactional leadership style	2.86 ± 1.05	46.90%
Organizational support	2.95 ± 0.96	48.75%
Employee empowerment	3.11 ± 0.91	52.85%

### Level of organizational commitment

In this study, among organizational commitment domains, the affective commitment dimension had the highest mean score (3.10 ± 0.93). The overall mean score for organizational Commitment was 2.95 ± 0.65 (%SM = 48.80% with 95% CI: 47.39, 50.24,) with 53.68% of health professionals scoring higher than the composite percentage sum mean score. (Table 4).

**Table 4:** Level of organizational commitment of health professionals working in public hospitals of SW Oromia, 2021 (n = 545)

	SD	D	N	A	SA	M (SD)	%SM	95% CI for %SM		Above %SM
								Lower	Upper	
**Overall organizational commitment**						**2.95 (0.67)**	**48.80**	**47.39**	**50.24**	**53.68**
**Continuance commitment**						**2.89 (0.79)**	**47.30**	**45.63**	**48.97**	**56.88**
I feel that I have very few options to consider leaving this organization	39	112	276	92	26	2.92 (0.92)	47.89	45.95	49.82	
One of the few serious consequences of leaving this organization would be the scarcity of available alternatives	45	141	239	95	25	2.84 (0.96)	46.05	44.03	48.07	
One of the major reasons I continue to work for this organization is that leaving would require considerable personal sacrifice and besides this, another organization may not match the overall benefit I have	44	112	260	100	29	2.92 (0.96)	48.07	46.05	50.09	
**Normative commitment**						**2.91 (0.89)**	**47.79**	**45.92**	**49.66**	**51.47**
I do not believe that a person must always be loyal to his or her organization	94	150	78	117	106	2.98 (1.40)	69.59	46.64	52.53	
Jumping from organization to organization does not seem at all unethical to me	91	168	90	147	49	2.81 (1.25)	45.18	42.55	47.82	
I do not think that to be a ‘company man’ or ‘company woman’ is sensible anymore	56	86	215	123	65	3.10 (1.24)	52.52	50.16	54.89	
It would not be too costly for me to leave my organization now	73	182	126	133	30	2.75 (1.13)	43.79	41.42	46.17	
**Affective Commitment**						**3.10 (0.93)**	**51.7**	**49.71**	**53.62**	**49.91**
I enjoy discussing my organization with people outside it	65	126	133	176	45	3.02 (1.17)	50.46	48.00	52.91	
It would be very hard for me to leave my organization right now, even if I wanted to	52	139	119	188	47	3.07 (1.15)	51.79	49.37	54.21	
Right now, staying with my organization is a matter of necessity as much as desire	34	146	146	165	54	3.11(1.09)	52.71	50.39	55.02	

**Notes**: SD: Strongly disagree; D: Disagree; N: Neutral; A: Agree; SA: Strongly Agree; M (SD): Mean with Standard Deviation; %SM: standardized percentage of mean score

### Factors associated with organizational commitment

In this study, a simple linear regression analysis was performed for all independent variables to see their independent effect on the level of organizational commitment. Accordingly, eight variables were identified as having less than 0.2 significance level in the bivariable simple linear regression and entered into the final multivariable linear regression model. The final model explained 77% (Adjusted R Square = 0.77) of the variability in the organizational commitment of healthcare professionals.

Job satisfaction-related factors: (work climate, recognition, supervisor support, and workload) was found to have a positive significant association with organizational commitment. Among these dimensions, work climate was the strongest predictor of organizational commitment. For a unit increase in work climate, the organizational commitment score increases by 0.32 units (95% CI: 0.18, 0.46).

Besides, there was a positive significant association between leadership styles and organizational commitment. In this regard, the organizational commitment score increases by 0.42 units for a unit increase in the transformational leadership style (95% CI: 0.28, 0.55). Similarly, for a unit increase in transactional leadership style, the organizational commitment score increases by 0.54 units (95% CI: 0.45, 0.62).

Employee empowerment was also found to have a positive significant association with organizational commitment. For a unit increase in employee empowerment, the organizational commitment score increases by 0.08 units (95% CI: 0.05, 0.11) (Table 5).

**Table 5:** Factors associated with organizational commitment of health professionals working in public hospitals of southwestern Oromia, 2021 (n = 545)

Variables	Unstandardized coefficient	Standardized coefficient	P-value	[95% CI for B]	
	B	β		Lower	Upper
Age	0.003	0.01	0.68	-0.01	0.02
Length of stay in current hospital	-0.002	-0.00	0.88	-0.03	0.03
Salary	-0.00	-0.07	0.05*	-0.00	5.84
Sex of participants					
Female	Ref	Ref			
Male	0.05	0.02	0.05*	-0.10	0.21
Educational Status					
Diploma	Ref	Ref			
Degree	0.26	0.08	0.18	-0.12	0.21
Masters degree and above	0.19	0.04	0.26	-0.14	0.53
Work experience in other facilities					
Having experience in other health facilities	Ref	Ref			
Not having experience in other health facilities	0.17	0.04	0.06*	-0.00	0.35
Type of position					
Managerial position	Ref	Ref			
Non-managerial position	0.00	0.00	0.96	-0.20	0.21
Living house					
Living inside the compound of a health facility	Ref	Ref			
Living outside the compound of the health facility	0.09	0.02	0.45	-0.15	0.34
Type of profession					
Doctor	Ref	Ref			
Laboratory professional	-0.16	-0.03	0.75	-1.16	0.83
Midwifery	-0.34	-0.07	0.04*	-0.68	-0.00
Nurse	-0.29	-0.08	0.06*	-0.61	0.01
Working unit or department					
Emergency	Ref	Ref			
IPD	-0.11	-0.03	0.40	-0.39	0.15
Pharmacy	0.76	0.13	0.08*	-0.09	1.62
Work climate	0.33	0.19	< 0.001**	0.19	0.47
Recognition	0.23	0.13	< 0.001**	0.12	0.33
Organizational support	0.13	0.02	0.09*	-0.02	0.28
Supervisor support	0.23	0.13	< 0.001**	0.13	0.33
Workload	0.27	0.16	< 0.001**	0.19	0.36
Transformational leadership style	0.42	0.24	< 0.001**	0.28	0.55
Transactional leadership style	0.54	0.31	< 0.001**	0.45	0.62
Employee empowerment	0.08	0.23	< 0.001**	0.05	0.11

Adjusted R square = 0.77; * Significant at p value ≤ 0.05; ** Significant at p value ≤ 0.001; max VIF = 3.2 (no multicollinearity VIF less than 10); CI = Confidence interval, IPD: Inpatient Department

## Discussion

In this study, the magnitude of organizational commitment in the southwestern Oromia public health facilities was 48.8%. Health professionals’ job satisfaction, leadership style, and employee empowerment are factors associated with organizational commitment.

Organizational commitment in the current study was lower compared to related studies conducted in Saudi Arabia [14], in Ethiopia Gurage zone [9], Bench-sheko zone [7], and Jimma university specialized hospital [17], where the percentage mean score of organizational commitment were 57.3%, 64.81%, 74.6%, and 88.06%, respectively. These differences could be due to socioeconomic, political, and cultural variations, particularly in the Saudi Arabia study, and participants’ regard to nurses alone at Jimma University specialized hospital. Besides, unlike the studies conducted at Gurage and Benchsheko Zone, which addressed health professionals working in health centers, our study was done in a hospital setting, where health professionals would face a large volume of patient flow and a high workload. Furthermore, during the COVID-19 pandemic, the medical environment is quite intensive. The combination of pressure, high vulnerability to COVID-19, spikes in workload, and a stressful workplace has produced a difficult organizational climate that may reduce health professionals’ commitment to their organization.

Our study suggested that satisfaction with work climate and recognition were significantly associated with organizational commitment. This implies that improving job satisfaction, specifically enhancing the working environment and recognition, is an important variable in promoting employees’ organizational commitment. This finding is in line with the result of other similar studies conducted in Ethiopia and Riyad [7, 9, 30]. This association can be explained by the fact that organizations with a conducive work environment will give healthcare professionals a sense of security and comfort, and this impression may intern lead employees to be committed to their organization, support its goals, and maintain membership [31]. Recognition of health professionals’ role and contribution to the organization may also have an impact on their perception of self-worth within the organization, and hence on their commitment to the organization.

In this study, satisfaction with supervisor support and workload were significantly associated with organizational commitment. This finding is in agreement with the study conducted in Nederland [32]. This can be explained by the fact that the supervisors may be regarded by their employees as representatives of the organization, and they may respond to the organization by expressing their attitudes or perception toward their supervisor.

Our finding also shows that leadership styles were found to have a significant positive association with the level of organizational commitment. This finding is congruent with the result of other similar studies conducted in Ethiopia and Saudi Arabia [7, 9, 14]. This can be explained by the characteristics of the transformational leadership style, which include idealized influence, inspirational motivation, intellectual stimulation, and individualized consideration, in which the leader gives followers a vision and a sense of mission and earns their respect and confidence from followers while also providing support, encouragement, and developmental opportunities.

When leaders exhibit transformational leadership behavior, employees express a highly personal connection with their leaders and go beyond self-interest because they are highly motivated and inspired to achieve what is expected of them which makes them committed [33].

In our study, employee empowerment was found to have a significant association with organizational commitment. This finding was in agreement with a study conducted in Egypt [34]. This may be the fact that employees who feel empowered at work are more likely to have a sense of autonomy or control over their work, believe they are competent in their skills and that they can make a difference. They are also strong, self-assured individuals, who are committed to a meaningful goal and show initiative to attain it.

### Limitations of the study

The use of a self-reporting questionnaire might lead to reporting bias, which may occur when respondents erroneously interpret questions. We also admit that COVID-19 social distancing measures may have affected responses. Furthermore, a qualitative method was not used to triangulate the data.

## Conclusion

The level of organizational commitment of health professionals working in southwestern Oromia region public hospitals was found to be low. Dissatisfaction with recognition, supervisor support, work climate, and high workload had a negative effect on organizational commitment. Additionally, practicing good transformational and transactional leadership styles was found to be a significant predictor of organizational commitment. Furthermore, employee empowerment showed a significant positive effect on organizational commitment.

Therefore, to increase the commitment of health professionals to their organization, hospital managers and healthcare policymakers should consider the factors pointed out in this study.

## Data Availability

Data will be available from the corresponding author on reasonable request.

## Abbreviations

HCOs: Healthcare Organizations
HRH: Human Resource for Health
HSTP: Health Sector Transformation Plan
HWs: Health Workers
HWF: Health Work Force
IRB: Institutional Review Board
JS: Job Satisfaction
LMIC: Low and Middle-Income Countries
OC: Organizational Commitment
OST: Organizational Support Theory
SET: Social Exchange Theory
SDG: Sustainable Development Goals
SSA: Sub-Saharan Africa
SW: Southwest
UHC: Universal Health Coverage and WHO:World Health Organization
